# Negatively charged amino acids at the foot-and-mouth disease virus capsid reduce the virion-destabilizing effect of viral RNA at acidic pH

**DOI:** 10.1038/s41598-020-58414-8

**Published:** 2020-02-03

**Authors:** Flavia Caridi, Silvia López-Argüello, Alicia Rodríguez-Huete, Elisa Torres, María J. Bustos, Rodrigo Cañas-Arranz, Miguel A. Martín-Acebes, Mauricio G. Mateu, Francisco Sobrino

**Affiliations:** Centro de Biología Molecular “Severo Ochoa” (CSIC-UAM), Cantoblanco, Madrid, Spain

**Keywords:** Biotechnology, Microbiology, Structural biology

## Abstract

Elucidation of the molecular basis of the stability of foot-and-mouth disease virus (FMDV) particles is relevant to understand key aspects of the virus cycle. Residue N17D in VP1, located at the capsid inner surface, modulates the resistance of FMDV virion to dissociation and inactivation at acidic pH. Here we have studied whether the virion-stabilizing effect of amino acid substitution VP1 N17D may be mediated by the alteration of electrostatic charge at this position and/or the presence of the viral RNA. Substitutions that either introduced a positive charge (R,K) or preserved neutrality (A) at position VP1 17 led to increased sensitivity of virions to inactivation at acidic pH, while replacement by negatively charged residues (D,E) increased the resistance of virions to acidic pH. The role in virion stability of viral RNA was addressed using FMDV empty capsids that have a virtually unchanged structure compared to the capsid in the RNA-filled virion, but that are considerably more resistant to acidic pH than WT virions, supporting a virion-destabilizing effect of the RNA. Remarkably, no differences were observed in the resistance to dissociation at acidic pH between the WT empty capsids and those harboring replacement N17D. Thus, the virion-destabilizing effect of viral RNA at acidic pH can be partially restored by introducing negatively charged residues at position VP1 N17.

## Introduction

Foot-and-mouth disease virus (FMDV) is the etiological agent of a highly contagious disease that affects livestock and world trade^1–3^. This virus is the type species of the *Aphthovirus* genus within the family *Picornaviridae*. The viral particle is composed of a single stranded positive-sense genomic RNA molecule of about 8.5 kb in length encompassed in a capsid conformed by 60 copies of each of the four structural proteins (VP1 to VP4)^4^. One copy of each VP make a protomer, five protomers make a pentamer, and 12 pentamers associate to form the capsid^5^. The pentameric subunits constitute intermediates of capsid assembly and disassembly^1,6,7^. Mild acidification of the FMDV virion results in capsid disassembly, which may be required for viral uncoating and genome penetration from endosomes^8,9^.

Vaccination is the main tool for FMD control in areas where this disease is enzootic. Current FMD vaccines are based on chemically inactivated, structurally preserved intact virions^10^ and viral particle integrity is essential for maintaining adequate immunogenicity of FMDV virions and capsids as capsid pentamers are poorly immunogenic^11^. FMDV empty capsids stand as a safe alternative for conventional vaccines that avoid the risks associated with handling infectious virus and could facilitate differentiation between infected and vaccinated animals^12,13^. However, FMDV virions and, to an even higher extent, empty capsids are quite prone to dissociation not only at acidic pH, but even at neutral pH and moderate temperatures. The thermal and chemical instability of FMDV empty capsids poses a serious problem for their ongoing development as novel FMD vaccines^14,15^.

Elucidation of the mechanisms subjacent to the stability of FMDV is relevant to understand virus physical properties that are essential for the virus infection cycle, as well as for vaccine improvement and development. A number of mutations that make FMDV virions and/or empty capsids more resistant to heat-induced dissociation into pentamers have been identified along the interpentamer interfaces^12,16–19^. Mutations that confer different levels of acid sensitivity to the FMDV virion have been found preferentially distributed in two different regions of the capsid: the N terminus of VP1 and the interpentameric interface^20^. Indeed, single amino acid substitutions in the viral capsid can enhance acid resistance by lowering the pH required for uncoating^21–25^. One of such replacements, N17D at the N terminus of VP1, is located within an internal capsid position^26,27^ and conferred increased resistance to acidic pH in FMDV isolates from three different serotypes^22,24,25^.

It has been shown that the increased resistance of the mutant VP1 N17D virion to acid-induced infectivity inactivation associates with its increased resistance to viral capsid dissociation into pentameric subunits^23^. Remarkably, N17D is a nearly isosteric replacement that introduces an additional negative charge per capsid protomer (60 additional charges in the viral particle). As an explanation for the mechanism of action of this substitution, it was first proposed that this replacement could destabilize the interaction between VP1 and the C terminus of VP4 within the same protomer by removing a hydrogen bond with the VP4 G78 residue of the same protomer^22^. In addition, in serotype C FMDV residue 17 of VP1 is located only about 15 and 8 Å, respectively, from H140 and H143 in VP3 of the same protomer. Protonation of these residues has been proposed to exert an electrostatic repulsion with the dipole of an alpha helix in the neighbouring pentamer, facilitating capsid dissociation within the acidic environment of the endosome^28,29^. In this context, it was hypothesized that the negative charge introduced in mutant VP1 N17D could partially neutralize the positive charge of the protonated VP3 H143, reducing the interpentamer repulsion at acidic pH and leading to increased resistance against acid-induced dissociation into pentamers^22^.

In addition to any putative effect of the VP1 N17D substitution on intracapsid interactions, its acid-stabilizing effect could be related also (or instead) to some change in capsid-RNA interactions. Some evidence obtained with FMDV^30,31^ and Coxsackie B3 virus^32^ indicates that not only capsid proteins determine the stability of these picornaviruses, but also the viral RNA could contribute to determine virion stability or instability at both acidic pH and increased temperature. However, the potential role in virion stability of RNA-capsid interactions had not been explored for these viruses. Here, we provide evidence for an involvement of electrostatic interactions and of the viral nucleic acid on the virion-stabilizing effect of the VP1 N17D amino acid replacement at acidic pH. The results indicate that changes in RNA-capsid interactions through introduction of charged residues at the capsid inner surface may modulate the stability of FMDV virions at acidic pH. Such modulation was not observed in FMDV empty capsids.

## Results

### The electrostatic charge of residue VP1 17 determines the resistance to acidic-pH in FMDV particles

To test the hypothesis of the involvement of electrostatic alterations in the resistance to acidic pH conferred by the neutral to negatively charged substitution N17D, different FMDV mutants harbouring substitutions that either introduced a positive charge (R, K), a negative charge (E, D) or preserved neutrality at residue 17 of VP1 (A) were engineered from plasmid pMT28 (WT)^33^. Infectious virus was recovered from BHK-21 cells transfected with the different *in vitro*-synthesized viral transcripts.

**Figure 1:**
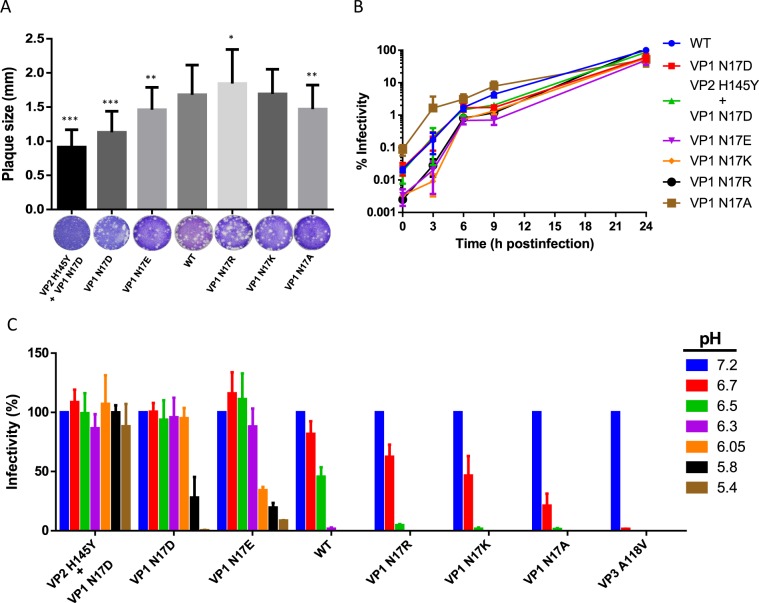
Growth characteristics and acidic resistance of FMDV mutants with substitutions at residue VP1 17. (**A**) Plaque size of FMDV mutants. BHK-21 cells were infected in agar semisolid medium, and viral plaques were detected by staining with crystal violet. About 100 viral plaques were analyzed for each virus. Asterisk/s denote statistically significant differences. (**B**) Single-step growth curve analysis of FMDV mutants. BHK-21 cells were infected (MOI of 1 PFU/cell), and the virus titer in the supernatants was determined by plaque assay at different hours postinfection. Results are expressed as the percentage of the virus titer relative to that of the WT at 24 h postinfection. Data are presented as means ± SD of triplicates. *P < 0.05; **P < 0.005; and ***P < 0.0001. (**C**) Acid sensitivity profiles of FMDV mutants. Equal PFU amounts of each of the viruses were treated (in triplicate) with different acid buffers (from pH 5.4 to 6.7) or pH 7.2 as a control. Samples were neutralized and plated on BHK-21 cell monolayers and the infectivity was calculated as the percentage of PFU recovered at each pH relative to that obtained at pH 7.2. Data are presented as means ± SD. The data for mutants VP1 N17D and VP1 N17E were confirmed in an independent experiment.

All the mutants were viable and produced infectious progeny virions. Minor variations in the plaque size of N17 mutants was observed, with mutants VP1 N17D, VP1 N17E and VP1 N17A showing a slight decrease in diameter (Fig. 1A) as that observed for VP1 N17D + VP2 H145Y, a highly acid-stable mutant selected by acidic incubation from mutant N17D^23^. Likewise, with the exception of the titers found at 3 h postinfection, no major differences were observed in the viral growth curves of the mutants relative to the WT virus (Fig. 1B).

Then, infectivity of these mutant viruses upon incubation in increasingly acidic pH medium was tested (Fig. 1C). Mutant VP3 A118V, selected in the presence of a NH_4_Cl, a lysosomotropic compound that blocks endosomal acidification, was included in this comparison as it exhibited a high acid sensitivity in comparison to WT^3^. Mutants VP1 N17E and VP1 N17D harbouring negatively charged residues showed a resistance to inactivation at acidic pHs (6.7 to 5.8) that was higher than that of the WT virus, as previously reported^22^, albeit they did not reach the resistance levels of double mutant VP1 N17D + VP2 H145Y whose infectivity was not affected even at a pH of 5.4. It should be noted also that mutant VP1 N17E is more resistant to pH 6.05 than mutant VP1 N17D, and this difference was confirmed in a new, independent experiment. Conversely, introduction at position VP1 17 of positively charged residues in mutants VP1 N17R and VP1 N17K rendered viruses with a decreased acidic resistance, as their infectivity was reduced by incubation at pH values lower than 7.2. Removal of the neutral amide group of VP1 N17 without introducing any charge (mutation VP1 N17A) resulted in the highest observed increase in the sensitivity of the virus to inactivation of infectivity at acidic pH (Fig. 1C), similar to that shown by mutant VP3 A118V^3^.

The increased resistance of mutant virions VP1 N17D and VP1 N17D + VP2 H145Y to acid-induced inactivation of their infectivity was previously shown to be caused by its increased resistance to dissociation into pentameric subunits^3,23^. As virion inactivation in these assays is known to be due to virion dissociation, measurements of virion inactivation provide a signature of virion dissociation. Thus, the observed changes in resistance to inactivation of infectivity in mutant virions with substitutions E, R, K or A at residue VP1 N17 are most likely mediated by changes in their resistance to capsid dissociation into pentamers.

To summarize, the truncation of amino acid VP1 N17 side chain by replacement to A or its replacement by positively charged residues (R or K) led to increased sensitivity of FMDV virions to its inactivation at acidic pH. In contrast, the replacement of VP1 N17 by negatively charged amino acids (D or E) increased the resistance of FMDV particles to acidic pH. These observations are consistent with previous results showing that introduction of the forward mutation D17G in the acid-resistant mutant VP1 N17D eliminated its resistance to acidic pH conferred by the N17D mutation; and that mutation VP1 Y18H that introduced a protonable side chain at a neighboring residue in the WT virus increased acid lability^20^. Taken together, these findings clearly indicate that the presence or the absence of electrostatic charge at residue VP1 17 has a role in controlling FMDV stability against inactivation at acidic pH, with a negative charge increasing stability.

### Electrostatic charge modifications at residue VP1 17 do not affect sensitivity to acidic pH of FMDV empty capsids

**Figure 2:**
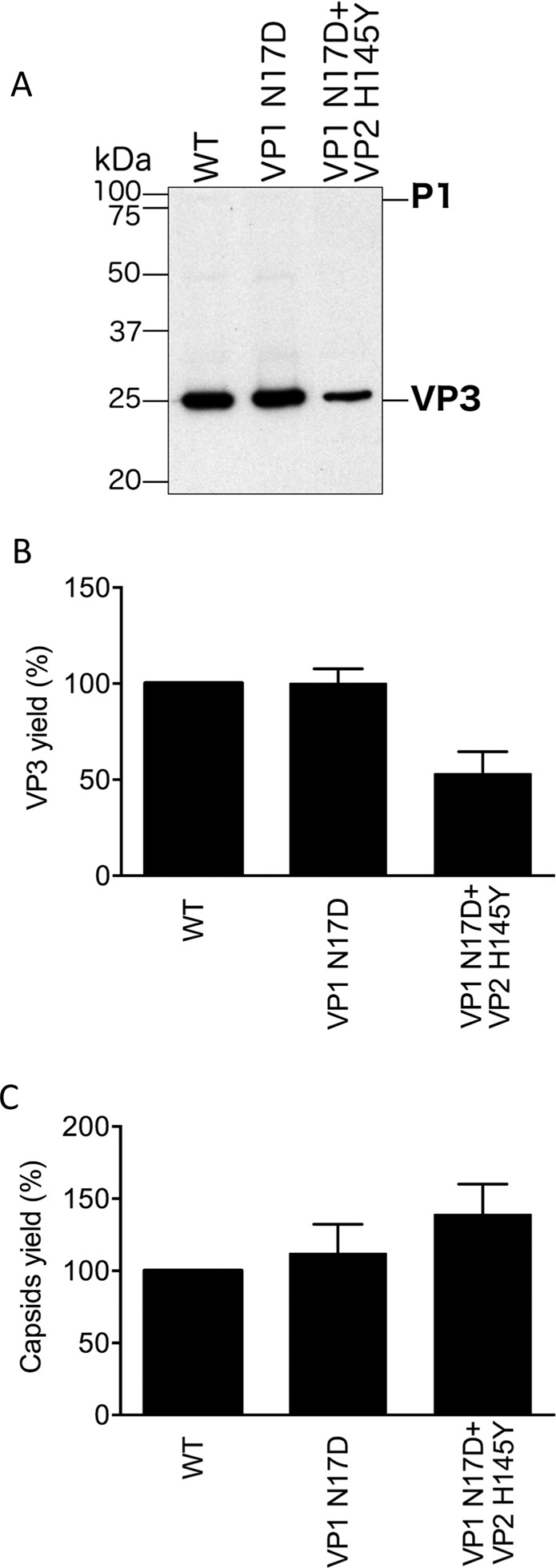
Expression and processing of capsid protein and capsid assembly of FMDV empty capsids with substitutions at residue VP1 17. Empty capsids were expressed, radiolabelled, and purified as previously described^16^. (**A**) Representative results of Western blot assays showing the expression and processing of capsid polyprotein P1 from WT and the mutant capsids indicated. M: molecular weight markers. The protein bands corresponding to the unprocessed polyprotein P1–2A and the end product VP3 (both determined by reactivity with an anti-VP3 antibody) are indicated. (**B**) Percentage yields of the mutant mature capsid protein VP3s relative to the WT control. The relative amount of radiolabelled empty capsids (mutants compared to wt) was estimated by densitometry of the corresponding bands. (**C**) Percentage yields of mutant assembled capsids relative to the WT control. The relative amount of radiolabelled empty capsids (mutants compared to WT) was estimated by determining the radioactivity associated to the 80 S peak observed by centrifugation in sucrose density gradients. For each mutant in (**B**) and (**C**), the averaged value obtained from two independent experiments and the corresponding error bar (SD) are shown.

We analyzed next the possibility that the virion-stabilizing effect of mutation VP1 N17D at acidic pH could require the presence of the viral nucleic acid inside the virion. Thus, recombinant empty capsids (WT and mutants VP1 N17D and VP1 N17D + VP2 H145Y) were produced in the absence of FMDV RNA by co-expression in BHK-21 cells of the P1–2A capsid precursor and the viral protease 3 C from plasmids pL1–1 and pSKRH-3C, respectively^34^. Previous evidence has unambiguously shown that in recombinant FMDV empty capsids VP0 is fully processed due to an RNA-independent, autocatalytic cleavage of VP0 to render VP4 and VP2^28,30^. In particular, the crystal structure of the empty capsid shows that cleavage and reveals that the empty capsid is virtually identical to the RNA-filled virion, even at atomic resolution, except for the absence of the RNA itself. The expression and processing of WT and mutant capsid proteins was compared by band densitometry of western blots probed with a capsid protein VP3-specific monoclonal antibody (Fig. 2A,B). In parallel, metabolically radiolabeled recombinant empty capsids VP1 17D, VP1 N17D + VP2 H145Y and WT were obtained under the same conditions indicated above and purified as previously described^16^.

The relative amount of processed protein (VP3) was similar for capsids VP1 17D and WT. A 50% reduction in the averaged relative amount of VP3 was observed between the double mutant and the WT which is close to the significance limits of the assay used. However, similar capsid yields were obtained for WT and the two mutants after the last purification step by centrifugation in sucrose gradients (Fig. 2C). To validate these differences between the WT and the double mutant in VP3 *versus* capsid yields, more precise measurements (which are very difficult to implement) would be required. If these differences were validated, a reasonable explanation could be based on the fact that capsid polyprotein P1 and capsid yields depend on different properties: the double mutation could make the P1 polyprotein (which contains VP3 before processing) somewhat less stable to degradation in the cell; however, this could be more than compensated if the same mutation increased the affinity between capsid subunits, leading to relatively higher capsid yields.

**Figure 3:**
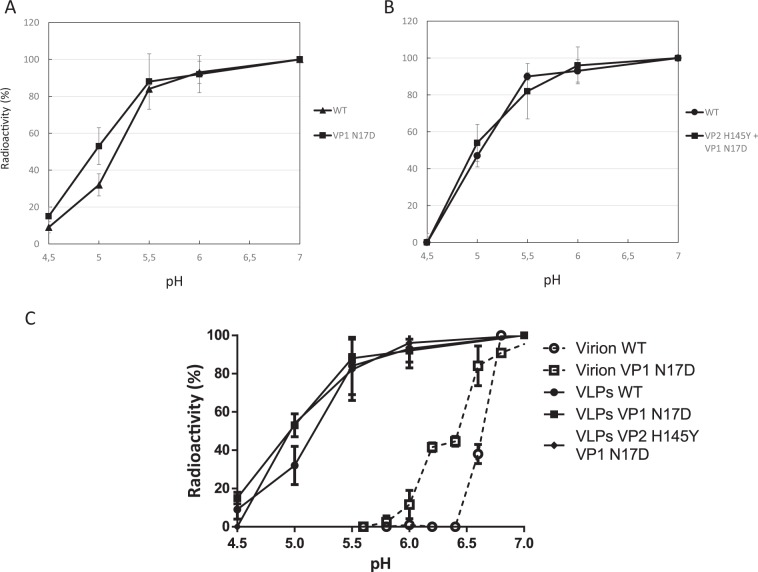
Analysis of empty capsid integrity after acid treatment. Acid sensitivity profiles of FMDV mutant empty capsids. Equal amounts of radiolabeled empty capsids (**A**) VP1 N17D (**B**) VP1 N17D + VP2 H145Y were treated with different acid buffers (from pH 4.5 to 7). Samples were neutralized and the amount of capsids at each pH was estimated by centrifugation in sucrose gradients and determination of the radioactivity present in each fraction (see Materials and Methods). In each experiment, the WT capsid was included as an internal control to compare stability in parallel under exactly the same conditions. The results obtained for each mutant capsid were normalized with respect to those for the WT capsid in the same experiment. The average values and error bars (SD) corresponding to two independent measurements are indicated. (**C**) Comparison of the acid sensitivity profiles of WT and capsid mutants VP1 N17D, VP1 N17D + VP2 H145Y (filled symbols) and those reported for the RNA-filled WT and mutant VP1 N17D virions^22^ (empty symbols).

Next, the stability against dissociation upon incubation in increasingly acidic pH medium was compared for WT and mutant empty capsids VP1 N17D and VP1 N17D + VP2 H145Y (Fig. 3A). Interestingly and contrary to what was observed with the infectivity of the RNA-filled virions (Fig. 1B), the WT and mutant empty capsids showed no significant differences in their stability at the range of pH tested (Fig. 3A,B). The WT capsids devoid of viral RNA showed a resistance to dissociation at acidic pH higher (~1.5 units) than that reported for the RNA-filled WT virions^3^ (Fig. 3C). A higher stability at acidic pH of the empty capsid relative to the virion had previously been observed for FMDV of a different serotype^28,30^.

To summarize these results, the presence of the viral RNA in the FMDV virion decreases its resistance against dissociation into subunits at acidic pH compared to the empty capsid. In turn, amino acid substitution VP1 N17D that introduce extra negative charges at the internal surface of the capsid increases the resistance of the virion, but not of the empty capsid, against dissociation at acidic pH, increasing the resistance of the virion against acid-induced inactivation of its infectivity.

## Discussion

Here we have demonstrated that replacement of amino acid residue VP1 N17 by a positively charged residue (K,R) or truncation of its side chain (A) decrease the resistance of FMDV virions to inactivation at acidic pH. In contrast, replacement of VP1 N17 by negatively charged residues (D,E), either alone or, in the case of VP1 N17D, accompanied by substitution H145Y, have the opposite effect. Interestingly, the stabilizing effect of the VP1 N17D and VP1 N17D + VP2 H145Y mutations at acidic pH was not observed in the absence of the viral RNA (by using empty capsids instead of virions).

It was previously shown that the presence of the viral RNA destabilizes the FMDV particle at acidic pH^30^ and the same has been found in this study using a different virus serotype (type C). These observations reveal that the viral RNA is a key factor for modulating (limiting) the pH stability of the FMDV virion at the acidic conditions found in the endosomes. This finding could indicate that RNA-capsid electrostatic interactions are governing uncoating of the FMDV genome in the cell.

The pH-dependent virus-destabilizing effect of the RNA could be explained as follows. Both at neutral and mildly acidic pH (as tested) the vast majority of the phosphate groups in the RNA and the carboxylate groups of aspartate and glutamate in the capsid will be unprotonated, and the vast majority of amino and guanidinium groups of lysine and argirine  will be protonated. However, due to their pKs the imidazole groups of histidines will largely shift from the unprotonated state at neutral pH to the protonated state at mildly acidic pH. The virion-destabilizing effect of the viral nucleic acid at acidic pH could be related, at least in part, to the protonation of histidine residues in the viral capsid. Protonation of His residues could neutralize the negative charge of Asp or Glu residues nearby, thus reducing the net electrostatic repulsion between the Asp and Glu residues in the capsid inner wall and the phosphates in the RNA. The higher stability of the empty capsid relative to the virion may be related to the removal of those repulsive capsid-RNA electrostatic interactions.

The structural basis for the stabilizing effect at acidic pH of substitution VP1 N17D in the presence of the RNA (virion) but not in its absence (empty capsid) is not straightforward either. We previously suggested that the mechanism of action of VP1 N17D could be related to the electrostatic neutralization of the positive charge of crucial histidine residues in VP3 that are protonated at acidic pH and trigger FMDV uncoating^22^. However, the results obtained here with empty capsids do not support this mechanism because, although protonation of these histidines occurs in the empty capsid^28^, the replacement VP1 N17D did not result in any significant change of its stability at acidic pH. This lack of effect of substitution VP1 N17D on empty capsid stability at acidic pH clearly indicates that the RNA within the capsid is mediating the virion-stabilizing effect of the VP1 N17D substitution. Interestingly, substitutions both VP1 N17D and N17E introduce 60 extra negative charges in the capsid inner surface, and so they could increase, not decrease, potential repulsive interactions between RNA and capsid in the virion. Thus, their virion-stabilizing effect must be based on a more complex structural effect that depends on the pH, the presence of the RNA and the charge at position VP1 17.

In rhinovirus, another picornavirus, it has been documented that in the native virion, genomic RNA interacts with VP2 and the VP1 N terminus, and that these interactions may vary during conformational rearrangements prior to RNA release^35^. The existence of direct interactions between residues located within the VP1 N terminus and the viral RNA may also explain why FMDV empty capsids displayed a more disordered VP1 N terminus than RNA-containing virions^36^. Residue VP1 17 is located at the VP1 N terminus. Thus, it is reasonable to propose that the charged carboxylates introduced in position 17 could lead to a rearrangement of the interactions between the 60 VP1 N-terminus and RNA segments, and these rearrangements could mediate the increased stability of the FMDV mutant virions VP1 N17D and N17E at acidic pH. Determination of the atomic structure of any of these mutant virions and comparison with the WT virion structure may provide structural support for this proposal.

This study suggests that certain amino acid residues in the FMDV capsid, such as the residue at position 17 of VP1, could be involved in preserving a biologically optimal compromise between efficiency of assembly and propensity for disassembly of the virion (reviewed in^37^), by modulating the destabilizing effect of the RNA. In at least some picornaviruses, likely including FMDV, the virion is assembled in a process that involves the coassembly of capsid and viral RNA^14,38^. A mutation like VP1 N17D could relieve the electrostatic repulsion between the RNA and the capsid proteins, increasing virion stability. However, such a mutation may not occur under normal conditions because it could impair virion disassembly by acidification in the endosomes during genome uncoating. Under the appropriate conditions (acidic medium), unless the virion is stabilized enough by reducing the repulsive effect of the RNA by a mutation such as VP1 N17D, the virion will lose its integrity. Under these unnatural conditions, a mutant such as VP1 N17D that would be normally present in the viral quasispecies in low proportion could be easily selected for.

In summary, the results indicate the virion-destabilizing effect at acidic pH of viral RNA can be partially restored by introducing negatively charged chemical groups at residue VP1 17. The increase in acid resistance of FMDV virions exerted by VP1 N17D and similar amino acid substitutions may be based on the modulation of certain RNA-capsid interactions. A precise knowledge of RNA–capsid interactions emerges as an important factor to understand FMDV stability and its importance during the infectious cycle.

## Materials and Methods

### Cells, viruses and infections

The origin of BHK-21 cells and culture procedures have been described^39^. The type C FMDV isolate, C-S8c1, and the mutants isolated from this virus VP1 N17D and VP1 N17D + VP2 H145Y have been described, as well as the procedures for virus infection and titration by plaque assay (in triplicate) in semisolid agar medium^23,40^. For single-step growth curve analysis of FMDV mutants, BHK-21 cells were infected (multiplicity of infection [MOI] of 1 PFU/cell), and the virus titer in the supernatants collected at different times postinfection was determined by plaque assay.

### Acid-induced FMDV inactivation assay

The assay was performed as described^3^. Briefly, triplicates of equal amounts of virus (PFU) were incubated at different pHs for 30 min at room temperature, and the remaining infectious virus was titrated in BHK-21 cells following pH neutralization.

### Nucleotide sequencing and site-directed mutagenesis of infectious cDNA clones

Reverse transcription of viral RNA its amplification by PCR and DNA sequencing were performed as described^3^. Nucleotide and amino acid positions are as those in FMDV C-S8c1 RNA (GenBank accession n°: AJ133357.1). Plasmid pMT28, containing the complete coding sequence of FMDV C-S8c1 RNA, was used to introduce selected nucleotide substitutions as reported^33,41^. Upon transfection of BHK-21 cells with the *in vitro*-synthesized viral transcripts harbouring each of the mutations, infectious viruses were recovered and the sequence corresponding to the capsid coding region of the viral RNA was sequenced as described^23^. This allowed to confirm the presence of the corresponding mutation in each of the viruses amplified, and to exclude reversions to the parental sequence or selection of further substitutions in the capsid-coding region of any of the viruses.

### Mutation, expression, radiolabelling and purification of FMDV empty capsids

To produce FMDV empty capsids, BHK-21 cells were infected with a recombinant vaccinia virus (VV), vTF7–3, that expresses the T7 RNA polymerase^42^, and infected cells were co-transfected with plasmids pL1–1 (containing the P1 capsid coding region of FMDV C-S8c1 plus the first 18 nucleotides of the 2A coding region, under the control of the T7 promoter), and pSKRH-3C (expressing the 3C protease of FMDV serotype A10)^43^. The corresponding point mutations were introduced in pL1–1 as described^34^ and their presence in the expressed viral proteins was confirmed by sequencing the capsid protein-coding region. Empty capsids were expressed, radiolabelled, and purified as previously described^16^.

### Analysis of stability of FMDV empty capsids against acid-induced dissociation

The stability of empty capsids against acid-induced dissociation was determined essentially as described^22^. Briefly, suspensions of radiolabeled empty capsids were incubated at different pHs (25 °C) and the amount of capsids was estimated, following pH neutralization, by sedimentation in sucrose gradients and counting the radiactivity at each fraction^22^. In each experiment, the WT capsid was included as an internal control to compare stability under the same experimental conditions. The results obtained for each mutant capsid were normalized relative to that of the WT capsid in the same experiment.

### Data analysis

Data are presented as mean ± standard deviation (SD) values. Analysis of variance (ANOVA) was performed using SPSS 15 (SPSS, Inc.). Bonferroni’s correction was applied for multiple comparisons. Statistically significant differences are indicated in figures by one asterisk for a *P* value < 0.05, two asterisks for a *P* value < 0.005 and three asterisks for a *P* value < 0.0001.
